# Supplementary material for: Pretreatment Adherence to a Priori-Defined Dietary Patterns Is Associated with Decreased Nutrition Impact Symptom Burden in Head and Neck Cancer Survivors
Source: Nutrients. 2021 Sep 9;13(9):3149. doi: 10.3390/nu13093149 (PMC8464702; doi:10.3390/nu13093149)
Supplement: Supplementary file 1 [file nutrients-13-03149-s001.zip › nutrients-1358527-supplementary.pdf]

Supplementary Materials

Table S1. Pearson Correlation Coefficients (*r*) matrix with the diet quality indices included in this analysis.

	AHEI-2010	aMED	DASH	Low-Carbohydrate
AHEI-2010	--			
aMED	0.73	--		
DASH	0.76	0.72	--	
Low-Carbohydrate	0.13	−0.08	−0.06	--

Table S2. Stratified ORs and 95% CI for associations between quintiles of *a priori* defined diet quality index scores with NIS symptom summary score ≥ 12 1-year post-diagnosis (*n* = 323).

BMI

Underweight/Normal Weight (*n* = 101)

Index	Q1	Q2	Q3	Q4	Q5	p_{trend}	$p_{Q5\text{-}Q1}$
AHEI-2010	1.00	1.14 (0.23–5.76)	0.46 (0.09–2.28)	0.34 (0.06–1.67)	0.31 (0.06–1.53)	0.09	0.16
aMED	1.00	0.34 (0.09–1.17)	0.35 (0.08–1.57)	0.34 (0.07–1.62)	0.72 (0.15–3.77)	0.30	0.68
DASH	1.00	0.35 (0.05–2.01)	0.04 (0.01–0.23) **	0.12 (0.02–0.7) *	0.12 (0.01–0.83) *	0.01 *	0.04 *
Low Carbohydrate	1.00	1.13 (0.28–4.54)	0.88 (0.16–5.13)	0.40 (0.09–1.70)	0.26 (0.05–1.29)	0.04 *	0.11

Overweight/Obese (*n* = 222)

Index	Q1	Q2	Q3	Q4	Q5	p_{trend}	$p_{Q5\text{-}Q1}$
AHEI-2010	1.00	0.90 (0.34–2.31)	1.13 (0.44–2.91)	0.40 (0.16–1.02)	0.71 (0.27–1.84)	0.19	0.49
aMED	1.00	0.31 (0.12–0.76) *	0.42 (0.17–1.05)	0.52 (0.22–1.20)	0.27 (0.09–0.76) *	0.05	0.01 *
DASH	1.00	0.87 (0.33–2.25)	0.48 (0.2–1.12)	0.58 (0.23–1.40)	0.57 (0.22–1.42)	0.14	0.23
Low Carbohydrate	1.00	1.50 (0.63–3.63)	1.10 (0.44–2.76)	0.77 (0.31–1.91)	1.68 (0.66–4.36)	0.62	0.28

Smoking Status

Current/Former Smokers (*n* = 224)

Index	Q1	Q2	Q3	Q4	Q5	p_{trend}	$p_{Q5\text{-}Q1}$
AHEI-2010	1.00	0.60 (0.21–1.65)	0.32 (0.11–0.87) *	0.20 (0.07–0.55) **	0.42 (0.14–1.18)	0.02 *	0.10

aMED	1.00	0.38 (0.15–0.95) *	0.26 (0.10–0.66) **	0.33 (0.13–0.81) *	0.40 (0.13–1.21)	0.04 *	0.10
DASH	1.00	0.78 (0.31–1.98)	0.25 (0.10–0.63) **	0.29 (0.11–0.75) *	0.50 (0.18–1.38)	0.02 *	0.18
Low Carbohydrate	1.00	1.28 (0.52–3.15)	1.15 (0.46–2.84)	0.51 (0.20–1.31)	1.31 (0.49–3.58)	0.82	0.59

Nonsmokers (*n* = 99)

**Index**	**Q1**	**Q2**	**Q3**	**Q4**	**Q5**	p_{trend}	p_{Q5-Q1}
AHEI-2010	1.00	1.90 (0.44–8.54)	2.62 (0.66–11.25)	0.94 (0.22–4.00)	1.11 (0.28–4.49)	0.86	0.89
aMED	1.00	0.64 (0.18–2.22)	1.34 (0.27–6.96)	0.37 (0.07–1.78)	0.41 (0.09–1.78)	0.21	0.24
DASH	1.00	0.32 (0.07–1.33)	0.79 (0.20–3.12)	0.29 (0.07–1.12)	0.41 (0.09–1.67)	0.18	0.22
Low Carbohydrate	1.00	1.72 (0.46–6.71)	0.4 (0.08–1.82)	0.81 (0.22–2.95)	0.46 (0.09–2.21)	0.23	0.34

Cancer Stage

Stages 0–2 (*n* = 104)

**Index**	**Q1**	**Q2**	**Q3**	**Q4**	**Q5**	p_{trend}	p_{Q5-Q1}
AHEI-2010	1.00	0.78 (0.19–3.14)	0.76 (0.17–3.37)	0.81 (0.19–3.48)	0.54 (0.1–2.66)	0.53	0.45
aMED	1.00	0.12 (0.02–0.57) *	0.12 (0.02–0.53) **	0.38 (0.09–1.46)	0.32 (0.05–1.80)	0.27	0.21
DASH	1.00	0.15 (0.03–0.61) *	0.34 (0.07–1.41)	0.19 (0.03–0.94) *	0.25 (0.04–1.35)	0.13	0.11
Low Carbohydrate	1.00	1.55 (0.40–6.17)	0.66 (0.17–2.52)	0.71 (0.14–3.38)	0.71 (0.17–2.91)	0.44	0.64

Stages 3,4 (*n* = 219)

**Index**	**Q1**	**Q2**	**Q3**	**Q4**	**Q5**	p_{trend}	p_{Q5-Q1}
AHEI-2010	1.00	0.64 (0.23–1.71)	0.89 (0.32–2.44)	0.29 (0.11–0.72) **	0.62 (0.23–1.63)	0.14	0.33
aMED	1.00	0.61 (0.23–1.61)	0.49 (0.20–1.12)	0.35 (0.11–1.13)	0.40 (0.14–1.15)	0.07	0.09
DASH	1.00	1.09 (0.37–3.24)	0.42 (0.16–1.06)	0.37 (0.14–0.92) *	0.48 (0.16–1.44)	0.02 *	0.19
Low Carbohydrate	1.00	1.43 (0.56–3.71)	1.30 (0.52–3.28)	0.70 (0.28–1.71)	1.32 (0.50–3.53)	0.85	0.58

HPV Status

Positive (*n* = 71)

**Index**	**Q1**	**Q2**	**Q3**	**Q4**	**Q5**	p_{trend}	p_{Q5-Q1}
AHEI-2010	1.00	0.06 (0.01–0.49) *	0.46 (0.06–3.25)	0.24 (0.02–1.73)	0.66 (0.07–5.23)	0.53	0.69
aMED	1.00	0.02 (0–0.24) **	0.05 (0–0.47) *	0.02 (0–0.76) *	0.20 (0.01–2.71)	0.41	0.26
DASH	1.00	0.18 (0.02–1.21)	0.4 (0.06–2.43)	0.19 (0.03–1.14)	0.34 (0.04–2.49)	0.31	0.29

Low Carbohydrate	1.00	6.66 (1.09–54.64)	12.28 (1.37–207.13) *	2.59 (0.5–15.25)	1.49 (0.27–8.67)	0.80	0.65

Negative (*n* = 92)

**Index**	**Q1**	**Q2**	**Q3**	**Q4**	**Q5**	p_{trend}	$p_{Q5\text{-}Q1}$
AHEI-2010	1.00	0.14 (0.02–0.68) *	0.36 (0.07–1.73)	0.21 (0.04–0.97)	0.72 (0.14–3.67)	0.90	0.69
aMED	1.00	0.42 (0.11–1.55)	0.66 (0.17–2.60)	0.72 (0.13–4.17)	0.8 (0.20–3.28)	0.99	0.76
DASH	1.00	0.48 (0.10–2.22)	0.27 (0.07–1.04)	0.48 (0.10–2.13)	0.48 (0.09–2.54)	0.27	0.39
Low Carbohydrate	1.00	2.23 (0.52–10.79)	0.68 (0.16–2.76)	0.36 (0.07–1.59)	1.04 (0.25–4.37)	0.57	0.96

Education

High School or Less (*n* = 91)

**Index**	**Q1**	**Q2**	**Q3**	**Q4**	**Q5**	p_{trend}	$p_{Q5\text{-}Q1}$
AHEI-2010	1.00	0.30 (0.04–1.85)	0.33 (0.04–2.17)	0.29 (0.04–1.88)	0.05 (0.01–0.32) **	<0.01 **	<0.01 **
aMED	1.00	0.17 (0.03–0.8) *	0.16 (0.03–0.89) *	0.23 (0.03–1.48)	0.01 (0–0.13) **	<0.01 **	<0.01 **
DASH	1.00	0.35 (0.05–2.18)	0.51 (0.09–2.78)	0.14 (0.02–0.72) *	0.14 (0.03–0.68) *	0.01 *	0.02 *
Low Carbohydrate	1.00	2.59 (0.48–16.76)	0.82 (0.17–4.02)	1.37 (0.28–7.1)	0.80 (0.16–4.06)	0.60	0.79

Some College or Greater (*n* =232)

**Index**	**Q1**	**Q2**	**Q3**	**Q4**	**Q5**	p_{trend}	$p_{Q5\text{-}Q1}$
AHEI-2010	1.00	0.96 (0.38–2.46)	0.67 (0.27–1.65)	0.77 (0.31–1.93)	1.05 (0.41–2.69)	0.90	0.92
aMED	1.00	0.48 (0.22–1.05)	0.71 (0.26–1.89)	0.97 (0.34–2.81)	0.71 (0.27–1.86)	0.77	0.48
DASH	1.00	0.36 (0.15–0.83) *	0.48 (0.18–1.26)	0.61 (0.24–1.53)	0.59 (0.21–1.62)	0.51	0.31
Low Carbohydrate	1.00	1.15 (0.48–2.74)	1.08 (0.44–2.64)	0.5 (0.20–1.21)	1.07 (0.42–2.77)	0.59	0.88

Tumor Site

Oral Cavity (*n* = 96)

**Index**	**Q1**	**Q2**	**Q3**	**Q4**	**Q5**	p_{trend}	$p_{Q5\text{-}Q1}$
AHEI-2010	1.00	0.71 (0.16–3.17)	1.19 (0.26–5.65)	1.09 (0.25–4.87)	1.05 (0.23–4.77)	0.76	0.95
aMED	1.00	0.15 (0.03–0.7) *	0.29 (0.05–1.34)	0.38 (0.08–1.62)	0.37 (0.06–1.97)	0.31	0.25
DASH	1.00	0.18 (0.03–0.82) *	0.77 (0.17–3.42)	0.35 (0.06–1.82)	0.19 (0.03–1.12)	0.34	0.08
Low Carbohydrate	1.00	1.36 (0.32–5.89)	0.59 (0.14–2.44)	0.81 (0.15–4.34)	0.66 (0.15–2.91)	0.44	0.59

Oropharynx (*n* = 157)

Index	Q1	Q2	Q3	Q4	Q5	p_{trend}	$p_{Q5\text{-}Q1}$
AHEI-2010	1.00	0.71 (0.19–2.54)	0.5 (0.14–1.66)	0.35 (0.1–1.17)	0.50 (0.14–1.72)	0.15	0.28
aMED	1.00	0.45 (0.14–1.35)	0.29 (0.07–1.06)	0.21 (0.05–0.94) *	0.29 (0.07–1.12)	0.04 *	0.08
DASH	1.00	0.56 (0.15–2.05)	0.17 (0.05–0.53) **	0.35 (0.09–1.28)	0.30 (0.08–1.11)	0.04 *	0.08
Low Carbohydrate	1.00	1.46 (0.46–4.87)	1.54 (0.50–4.97)	0.97 (0.35–2.73)	0.63 (0.20–2.00)	0.38	0.43

Larynx (*n* = 66)

Index	Q1	Q2	Q3	Q4	Q5	p_{trend}	$p_{Q5\text{-}Q1}$
AHEI-2010	1.00	0.80 (0.10–6.23)	0.40 (0.05–3.07)	0.18 (0.02–1.19)	0.12 (0.01–0.95)	0.01 *	0.06
aMED	1.00	0.45 (0.09–2.18)	0.32 (0.04–1.92)	0.05 (0.00–0.63) *	0.38 (0.04–2.89)	0.12	0.36
DASH	1.00	6.40 (0.64–160.43)	0.16 (0.02–1.02)	0.27 (0.04–1.65)	1.13 (0.13–10.3)	0.37	0.91
Low Carbohydrate	1.00	0.83 (0.10–6.63)	0.63 (0.11–3.56)	0.39 (0.03–3.44)	1.73 (0.26–12.95)	0.74	0.58

Treatment Modality

Treatment Involving Radiation as Primary or Adjuvant Modality (*n* = 232)

Index	Q1	Q2	Q3	Q4	Q5	p_{trend}	$p_{Q5\text{-}Q1}$
AHEI-2010	1.00	0.93 (0.35–2.48)	0.79 (0.30–2.07)	0.38 (0.15–0.98) *	0.64 (0.24–1.67)	0.13	0.36
aMED	1.00	0.66 (0.26–1.67)	0.73 (0.32–1.62)	0.52 (0.16–1.73)	0.43 (0.16–1.16)	0.10	0.10
DASH	1.00	1.44 (0.53–3.94)	0.59 (0.23–1.47)	0.59 (0.23–1.53)	0.57 (0.22–1.44)	0.07	0.24
Low Carbohydrate	1.00	1.51 (0.59–3.97)	1.14 (0.48–2.70)	0.69 (0.30–1.58)	1.71 (0.65–4.73)	0.81	0.29

Treatment Not Involving Any Radiation (i.e., Surgical or Chemotherapy Alone) (*n* = 91)

Index	Q1	Q2	Q3	Q4	Q5	p_{trend}	$p_{Q5\text{-}Q1}$
AHEI-2010	1.00	0.76 (0.16–3.53)	0.26 (0.05–1.21)	1.5 (0.31–7.32)	0.38 (0.06–2.03)	0.46	0.27
aMED	1.00	0.08 (0.02–0.34) **	0.17 (0.02–0.93) *	0.23 (0.04–1.11)	0.53 (0.08–3.10)	0.21	0.49
DASH	1.00	0.24 (0.05–0.97)	0.18 (0.03–0.82) *	0.09 (0.01–0.47) **	0.49 (0.09–2.61)	0.15	0.4
Low Carbohydrate	1.00	2.73 (0.68–11.78)	1.14 (0.27–4.91)	0.48 (0.09–2.38)	0.33 (0.06–1.59)	0.08	0.18

* $p < 0.05$

** $p < 0.01$

All models adjusted for age, cancer stage, smoking status, HPV status, total calories, and baseline NIS symptom summary score. Covariates for which models were stratified were not included in the corresponding model.

Table S3. ORs and 95% CI for associations between quintiles of *a priori* defined diet quality index scores with NIS symptom summary score ≥ 12 1-year post-diagnosis stratified on the presence of significant pretreatment NIS.

NIS Present at Study Entry (n = 251)

**Index**	**Q1**	**Q2**	**Q3**	**Q4**	**Q5**	p_{trend}	p_{Q5-Q1}
AHEI-2010	1.00	0.68 (0.27–1.67)	0.62 (0.25–1.52)	0.34 (0.13–0.82) *	0.57 (0.23–1.39)	0.09	0.22
aMED	1.00	0.40 (0.16–0.97) *	0.41 (0.18–0.91) *	0.51 (0.22–1.15)	0.50 (0.19–1.33)	0.23	0.17
DASH	1.00	0.39 (0.15–0.96) *	0.27 (0.11–0.63) **	0.33 (0.13–0.81) *	0.40 (0.15–1.04)	0.05	0.06
Low Carbohydrate	1.00	1.39 (0.60–3.28)	0.88 (0.38–2.07)	0.54 (0.23–1.25)	1.02 (0.42–2.47)	0.45	0.97

NIS Not Present at Study Entry (n = 72)

**Index**	**Q1**	**Q2**	**Q3**	**Q4**	**Q5**	p_{trend}	p_{Q5-Q1}
AHEI-2010	1.00	1.02 (0.15–7.16)	0.79 (0.12–5.24)	0.44 (0.07–2.51)	0.78 (0.11–5.34)	0.52	0.79
aMED	1.00	1.59 (0.19–18.36)	0.16 (0.02–0.81) *	0.20 (0.03–1.32) [a]	NA	0.11	0.10 [b]
DASH	1.00	0.70 (0.10–4.64)	0.28 (0.04–1.81)	0.13 (0.01–0.98)	0.24 (0.03–1.72)	0.06	0.17
Low Carbohydrate	1.00	2.62 (0.34–24.15)	2.95 (0.52–19.25)	1.53 (0.19–13.05)	1.64 (0.23–13.24)	0.67	0.63

*$p < 0.05$

**$p < 0.01$

All models adjusted for age, cancer stage, smoking status, total calories, and HPV status.

[a] Quartiles were used in modeling given the significant amount of ties when attempting to fit quintiles.

[b] Reflects p_{Q4-Q1}
